# Correlated metadiscourse and metacognition in writing research articles: A cross-linguistic and cross-cultural study

**DOI:** 10.3389/fpsyg.2022.1026554

**Published:** 2022-11-14

**Authors:** Fei-Hong Gai, Yao Wang

**Affiliations:** Department of English College, Ocean University of China, Qingdao, China

**Keywords:** metadiscourse, culture-specificity, language-specificity, metacognition, mediation, monitoring

## Abstract

Metadiscourse represents a producer’s intention to guide a receiver’s interpretation of the textual meanings. It is a highly dynamic topic in discourse analysis and language education. Related studies provide a way to understand language in use, and contribute to a better understanding about the relationship between the seemingly unconscious language choices and the social contexts. Based-on a corpus of 150 research articles (RAs) written by English L1 scholars, Chinese ESL scholars and Chinese L1 scholars, this study compared their interactive and interactional metadiscourse strategies cross-linguistically and cross-culturally. Quantitative results manifest significantly higher metadiscursive frequencies in English-medium RAs than in Chinese-medium RAs, and significantly higher metadiscursive frequencies in RAs written by British-American scholars than by Chinese scholars. Also, Chinese ESL writers reveal L1-based transfer of discourse conceptualization. Apart from providing with cultural explanations, this study then particularly discusses cognitive implications of culture-specific and language-specific metadiscourse variations by addressing the connections between metacognition and metadiscourse. With the proposed Model of Correlated Metadiscourse and Metacognition, it argues that metadiscourse is the linguistic reflection of metacognition and that metacognition exerts mediation and monitoring over cognitive objects partly by the means of metadiscourse.

## Introduction

Academic writing is widely conceptualized as a self-regulated process which entails “[the] production of thought for oneself or others under the direction of one’s goal-directed metacognitive monitoring and control, and the translation of that thought into an external symbolic representation” (Hacker et al., 2009, p. 154). On the other side, as Martin-Martin (2008) contends, academic writing not simply constructs content and information, but imposes conviction and influence on readers, and connects texts with disciplines through making linguistic choices. In this process of applied metacognition, metadiscourse helps to re-establish the significance of interpersonal aspects of language (Jiang and Hyland, 2022), and offers an approach to understanding the ways writers project themselves into the texts as they manage their communicative intentions. Seeking to fulfil writer-reader communication in research articles (RAs), it secures authorial positions, multiplies textual readability, and aligns writers with the text and readers (Hyland, 1998a). Related studies have pointed out how writers in academic communities negotiate with readers, draw closer different academic bodies, and establish academic identities and social relationships.

This paper attempts to propose an advancement of a cross-disciplinary theoretical framework that might help research in writing and discourse analysis from the perspective of correlated metadiscourse and metacognition. Based on a corpus analysis, it tentatively establishes a necessary link between cognitive aspects of discourse construction and the contexts to which they are related.

## Literature review

### Metadiscourse in RAs

Metadiscourse is defined as “[the] self-reflective expressions used to negotiate interactional meanings in a text, assisting the writer (or speaker) to express a viewpoint and engage with readers as members of a particular community” (Hyland, 2005, p. 37). William (1981), Vande Kopple (1985), Crismore et al. (1993), Hyland (2005), Ädel (2006) and other scholars conceptualize and classify metadiscourse from varying perspectives, but recent years have witnessed great influences by Hyland’s (2005) scheme which categorizes metadiscourse into interactive and interactional types. The former is to constrain and shape the discourse in the light of readers’ particular needs and expectations, and it is further divided into transitions, code glosses, frame markers, endophoric markers, and evidentials; the latter is aimed at making the writer’s view explicit and involving readers, and it is further differentiated into hedges, boosters, attitude markers, self-mentions, and engagement markers (see Table 1). The current study adopts Hyland’s conceptualization due to its clarity, inclusiveness and dynamism. Moreover, it takes a functional approach to metadiscourse using small clauses as the unit of analysis, which conveniently accommodates Chinese discourse whose sentences are not set borders grammatically but topically (Chao, 1955).

**Table 1:** An interpersonal model of metadiscourse (Hyland, 2005, p. 49).

Category	Function	Examples
**Interactive**	**Help to guide the reader through the text**	**Resources**
Transitions	Express relations between main clauses	In addition; but; thus; and
Frame markers	Refer to discourse acts, sequences or stages	Finally; to conclude; my purpose is
Endophoric markers	Refer to information in other parts of the text	Noted above; see figure; in “Literature Review”
Evidentials	Refer to information from other texts	According to X; Z states
Code glosses	Elaborate propositional meaning	Namely; such as; in other words
**Interactional**	**Involve the reader in the text**	**Resources**
Hedges	Withhold commitment and open dialogue	Might; perhaps; possible; about
Boosters	Emphasize certainty and close dialogue	In fact; definitely; it is clear that
Attitude markers	Expresses writers’ attitude to proposition	Unfortunately; I agree; surprisingly
Self mentions	Explicit reference to author(s)	I; we; my; me; our
Engagement markers	Explicitly build relationship with reader	Consider; note; you can see that

Metadiscourse is a well-acknowledged approach to the discourse analysis of RAs (Hyland and Jiang, 2022). On account of its roles in marking discourse structure, conveying writers’ attitude and engaging with readers, this paper understands metadiscourse as linguistic cues that writers consciously or unreflectively deploy as a response to their evaluation of readers’ need for elaboration and involvement. Accordingly, metadiscourse strategies refer to writers’ explicit set of linguistic choices that index their assumption of readers’ need to mark connections between propositions, their evaluation of readers’ potential reaction to related claims, and their anticipation of the appropriate way they can project themselves into the discourse as authoritative and credible insiders. That is, they are the rhetorical preferences that writers resort to as an assistance in the process of presenting research findings, securing understandings and acceptance of propositions, projecting themselves and readers, and negotiating social relations in ways that are accepted and valued by a specific discourse community. In practice, metadiscourse strategies are formed and developed based on writers’ estimation of how best they can help readers process the coded knowledge and comprehend what they are proposing. In a word, these strategies can be understood as “[a] recipient design filter” (Hyland, 2017, p. 158) assisting in spelling out the intended information by providing a commentary on it. The successful management of these disciplinarily-specific and generically-situated rhetorical strategies help to construct both knowledge and identities. Thereby, writers’ further adaption and adjustment also reveal how well they understand the community norms and values. Accordingly, metadiscourse strategies are simultaneously reflections of writers’ familiarity with readers and their adaption to the discourse community. And in fact, these strategies connect discourse with community. The seemingly unconscious deployment of metadiscourse strategies are nurtured through writers’ conscious participation into disciplinary conventions and activities that tie members of a specific discourse community into shared beliefs and practices. That’s why this study claims metadiscourse strategies employed by British-American and Chinese scholars index their respective cultural and rhetorical contexts.

### Metadiscourse as intersubjectivity-constructing resources

Studies in Corpus Linguistics and Discourse-functional Linguistics (e.g., Biber et al., 1999; Downing, 2001) acknowledge that certain linguistic resources not merely convey a subjective stance toward a following clause or utterance, but also emerge in a specific socioculturally-situated context. They can be used to construct agreement and affiliation (Chafe, 1986), to show disagreement and conflict with previous texts or between writers (Kärkkäinen, 2006), or to establish and maintain relationships between writers and readers (Hunston, 2007). More specifically, these linguistic devices can be utilized for writers to express their intersubjective and dialogical intentions. And this paper proposes that metadiscourse is an intersubjective, cognitive and social act.

Intersubjectivity is defined by Traugott (2003) as writers’ indirect manipulation of the interactants (i.e., writers and readers) involved in the academic activity. It is the way in which natural languages provide for the locutionary agent’s expression of his/her awareness of the addressee’s attitudes and beliefs. It is also the explicit expression of the speaker’s/writer’s attention to the *self* of addressees in both an epistemic sense (underscoring their presumed attitudes to the content of what is said), and a more social sense (highlighting their *face* or *image needs* associated with social stance and identity). This definition has at least three implications: (1) intersubjectivity involves writer-reader relationships in the writing events; (2) such relationships could be manipulated by writers through linguistic devices; and (3) such manipulation is not only decided by writers and readers but by linguistic and cultural factors.

Interestingly, metadiscourse strategies correspond to the three implications of intersubjectivity as follows: (1) they project relationships between writers and readers; (2) such relationships could be manipulated by writers through (but not limited to) metadiscourse devices; and (3) such manipulation is largely decided by linguistic and cultural factors. Metadiscourse construes positive and negative evaluation in RAs. The construal process reaches beyond the propositional meaning of texts and realizes the interactional and sociopragmatic functions. In practice, metadiscourse devices are specifically-defined and dynamically-changed functions and parameters. They are not only intersubjectivity-constructing resources in academic texts, but gain us insight into the potential factors that might influence this construction process. That is, we could explore how writers establish interpersonal relationship and construct intersubjectivity with predicted readers by the means of metadiscourse strategies which are subject to different cultures, communities, disciplines and genres.

Admittedly, the pragmatics of academic metadiscourse (Hyland, 1998b) and the relevance of pragmatics to writings are obvious in that writers interact with readers drawing on and manipulating institutionally-defined features and culturally-inscribed understandings. Pragmatics provides researchers with ways to analyze how writers encode ideas for readers and how readers decode writers’ intended meanings in particular communities. Actually, metadiscourse contributes to not merely making ideas understood (an illocutionary effect) but also accepted (a perlocutionary effect). Indeed, these features mark considerations for readers and appeal to common grounds based on shared professional and personal relationships.

Previous literature is predominantly interested in metadiscourse strategies by writers speaking different languages. While more investigations are devoted to English texts written by writers from different cultural backgrounds (e.g., Abdollahzadeh, 2011; Lee and Deakin, 2016), fewer studies have sought to explore texts written by English native-speakers compared to those by native-speakers of other languages (e.g., Mu et al., 2015; Li and Xu, 2020). However, they have hardly compared metadiscourse simultaneously across cultures and across languages. Contrastively, the current study ambitiously explores the cross-linguistically/culturally pragmatic influence at the discursive level.

### Metacognition in writing

Metacognition and appropriate strategy application have been identified as important components of skillful writing (Hacker et al., 2009). Flavell (1979, p. 906) defines metacognition as “[knowledge] about cognition and cognitive phenomena.” He further classifies the monitoring of cognitive enterprises that occur among four classes of phenomena: (1) metacognitive knowledge, (2) metacognitive experiences, (3) goals (or tasks), and (4) actions (or strategies). Among them, metacognitive knowledge refers to segment of one’s stored world knowledge that deals with people as cognitive creatures and that addresses their diverse cognitive tasks, goals, actions, and experiences. Metacognitive experiences are conscious, cognitive or affective experiences that accompany and pertain to any intellectual enterprise. Goals (or tasks) refer to the objectives of a cognitive enterprise. And actions (or strategies) are the cognitions or other behaviors employed to achieve goals.

Hacker et al. (2009) propose a Metacognitive Model of Writing in which the metacognitive process of writing is composed of two or more specifically related levels—an object-level and a meta-level. While the object-level engages cognitions about external stimuli (e.g., comprehending a text) or internal stimuli (e.g., setting goals), the meta-level includes cognitions concerning one’s object-level cognitions such as metaperception, metacomprehension, metamemory and metafantasizing. They argue that a lower level cognition (i.e., cognition occurring at the object-level) can be the subject of a higher level cognition, and cognitions at these two levels can occur simultaneously. In words, any thought can become the object of another thought, and theoretically, one’s cognitions of his/her meta-level cognitions can occur. In this model, the direction of flow of information between the two levels of metacognition is composed of two relations—monitoring and control. Metacognitive monitoring refers to awareness of one’s current thoughts or behavior, and it occurs when the meta-level is informed of cognitions happening at the object-level through the information flow from the object-level to the meta-level (Kluwe, 1982). Contrastively, metacognitive control means the modification of one’s current thoughts or behavior, and it occurs when the information flow intends to guide and direct object-level cognitions. In the term of Hacker (2018, p. 2), “[the] goal of control strategies is to produce thoughts and the goal of monitoring strategies is to observe the production of thoughts.” In effect, metacognitive control and monitoring have been recognized as being critical to writing for almost 40 years (e.g., Hayes and Flower, 1980; Bruer, 1997; Kellogg, 1999). Tentatively, the current study aims to highlight the meaning of metadiscourse as the linguistic reflection of metacognition, and the way metacognition exerts mediation and monitoring over cognitive objects partly by the means of metadiscourse.

Previous literature has hardly explained cross-cultural/linguistic variations of metadiscourse strategies in terms of cognitive implications nor has discursively extended the discussion to cognitive influence of L1 over L2. Although some of them do tentatively include cognition as an origin of the different metadiscourse strategies (e.g., Guillem, 2009; Golmohammadi et al., 2014), their understanding of cognition is mostly a socio-cognitive approach in which the cognition itself mainly means writers’/readers’ social awareness of the interaction between them. Studies in direct relation to cognition and Cognitive Linguistics are comparatively insufficient. The current paper attempts to make up some of the gaps by including cognitive implications of the potential metadiscursive uniformities and variations at the discursive level.

## Materials and methods

### Design of the study

**Figure 1:**
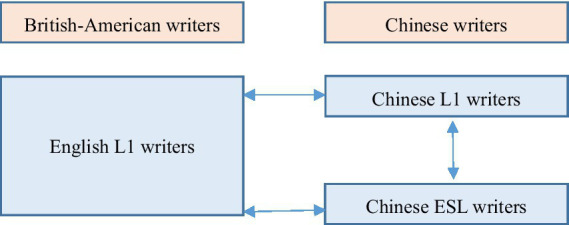
Three-way comparison of metadiscourse strategies in QERAs.

The comparative and contrastive methods were employed to locate trends and differences in the use of metadiscourse by English L1 scholars, Chinese ESL scholars and Chinese L1 scholars. Figure 1 below plots the three-way comparison under investigation. As can be seen, it was a both cross-linguistic and cross-cultural study. It was cross-linguistic in that it compared the metadiscourse strategies written in English and Chinese. It was cross-cultural since it compared metadiscourse strategies by English L1 writers to those by Chinese ESL writers. Importantly, cross-cultural and cross-linguistic differences under investigation did not exclude each other absolutely, but their most typical characteristics were taken here. Namely, languages were separated from cultures in this study because a particular language could describe diverse cultural events and a specific cultural event could be expressed by different languages (Wen, 2016). However, the current study admits that languages always take cultural conventions when depicting the cultural events, and that linguistic traits are frequently supposed to be in line with the established values of the communities in which languages are used.

### Corpus construction

To exclude potential influences of disciplines and research paradigms (Cao and Hu, 2014), a corpus of quantitative Economics research articles (QERAs) was compiled: Chinese-Native-Expert Chinese Corpus (CNECC), 50 Chinese QERAs by Chinese L1 scholars; Chinese-Native-Expert English Corpus (CNEEC), 50 English QERAs by Chinese ESL scholars; and English-Native-Expert English Corpus (ENEEC), 50 English QERAs by British/American scholars whose L1 is English. All QERAs were randomly selected from those published from 2014 to 2017 in prestigious national Chinese-medium journals (included in Chinese Social Sciences Citation Index) and international English-medium journals (included in Social Science Citation Index). To verify the authors’ L1, professional websites were visited, and inquiry mails were sent when necessary. Table 2 above presents details of the corpora.

**Table 2:** Description of the sub-corpora.

	CNECC	CNEEC	ENEEC
No. of RAs	50	50	50
No. of source journals	4	9	7
Length of texts	8,118–17,252	8,116–15,087	7,489–18,823
Average length of RA	12,365	10,295	13,661
Total no. of words	618,230	514,742	683,058

In view of the potential influence of research paradigm (Lincoln et al., 2018), which might result in different rhetorical choices and divergent metadiscourse markings, only quantitative RAs were included in the current corpus. To exclude the paradigmatic influence, sections about the research design, data collection and data analysis were reviewed to clarify the paradigmatic orientation which generally followed standard and typical Introduction–Method–Results–Discussion pattern or Introduction–Literature Review–Method–Results and Discussion–Conclusion pattern of empirical RAs (Lin and Evans, 2012). Full-length of original RAs were extracted from the selected journals. Following previous studies, this paper excluded titles, abstracts, information of authors, tables, figures, stand-alone quotations, references and acknowledgements.

### Data collection

This paper adopted Hyland’s (2005) metadiscourse scheme and followed a corpus-based methodology by analyzing and comparing the occurrence frequency of metadiscourse features among the three sub-corpora. First, it examined previous literature, and made a list of possible tokens of each type/sub-type of features. Following Hyland (*ibid.*), some controversial items (e.g., the intra-sentential subordinators) were kept for the functional approach of this study. Namely, judgement was not made according to whether items were for syntactic purpose or not. Then, some RAs were carefully scanned and read in search of potential metadiscourse, and the newly-found tokens were added into the list and the coding scheme. What’ more, footnotes, notes and appendix were included since they were importantly informative in QERAs. But printing features were excluded due to potential scanning mistakes. Next, all RAs were carefully scanned, read, and manually annotated in Microsoft Word. Every single metadiscourse feature was coded and identified in its context, and then all the possible tokens were added to the list. To ensure data reliability, five QERAs from each sub-corpus were independently annotated by three raters who were Chinese PhD candidates majoring in English Applied Linguistics and who received training for the data coding within the current coding scheme. The Kappa Statistic (Cohen et al., 2018) was applied to assess the inter-rater agreement for these features, and the results ranged from 0.72 to 1.0, revealing a mean of 0.87 for all categories and implying good reliability.

### Data analysis

The quantitative analysis was implemented to examine cross-cultural (ENEEC vs. CNEEC) and cross-linguistic (CNECC vs. CNEEC) effects on the frequency (per 10,000 words) of interactive and interactional metadiscourse. Following previous studies on metadiscourse in RAs, a Chi-square analysis was conducted to determine whether the differences were significant or not. The significance level was set at <0.05, <0.01 and <0.001.

## Results

Holistically, British-American scholars used more metadiscourse device (1152.61 tokens per 10,000 words in ENEEC) than Chinese scholars, and Chinese scholars publishing in the international community used more metadiscourse device (1040.06 tokens per 10,000 words in CNEEC) than those publishing in the domestic community (470.42 tokens per 10,000 words in CNECC). On the other side, writers of QERAs use more interactive features than interactional features. Interactive metadiscourse features were 607.01, 588.84 and 294.26 tokens per 10,000 words in ENEEC, CNEEC and CNECC, respectively, whereas interactional metadiscourse features were 545.61, 451.22 and 176.16 tokens per 10,000 words correspondingly. The distributions of interactive and interactional features in each sub-corpus were significantly different from each other, i.e., *p* < 0.001, Chi-square value = 237.08 in ENEEC; *p* < 0.001, Chi-square value = 988.79 in CNEEC; and *p* < 0.001, Chi-square value = 1876.99 in CNECC.

### Interactive metadiscourse

The descriptive statistics for interactive metadiscourse were summarized in Table 3 (per 10,000 words). The vertical comparison showed distribution consistency among the three sub-corpora. Generally, transitions occurred the most frequently, which was in accordance with previous findings (e.g., Hyland and Tse, 2004). What followed in orders was code glosses, endophoric markers, evidentials, and finally frame markers. Horizontally, these features manifested statistically significant differences both cross-linguistically and cross-culturally (CNEEC vs. CNECC: *p* < 0.001, Chi-square value = 5948.21; and ENEEC vs. CNEEC: *p* < 0.001, Chi-square value = 17.20). British-American economists and Chinese economists, consciously or unconsciously, made judgements of readers’ logical needs to markedly different degrees.

**Table 3:** Frequencies of metadiscourse in CNECC, CNEEC, and ENEEC.

	CNECC		vs.	CNEEC		vs.	ENEEC	
	Raw no.	10 *t*	Value of *p*	Raw no.	10 *t*	Value of *p*	Raw no.	10 *t*
**Interactive**								
Transitions	7,268	117.56	<0.001	12,202	237.05	<0.001	18,336	268.44
Code glosses	3,934	63.63	<0.001	7,015	136.28	0.009	9,696	141.95
Frame markers	1,384	22.39	<0.001	1738	33.76	0.207	2,215	32.43
Endophoric markers	3,646	58.97	<0.001	6,013	116.82	0.703	8,031	117.57
Evidentials	1960	31.7	<0.001	3,342	64.93	<0.001	3,184	46.61
Total	18,192	294.26	<0.001	30,310	588.84	<0.001	41,462	607.01
**Interactional**								
Hedges	4,386	70.94	<0.001	8,533	165.77	<0.001	13,537	198.18
Boosters	2,872	46.46	<0.001	5,544	107.7	<0.001	8,379	122.67
Attitude markers	2,118	34.26	<0.001	3,310	64.3	<0.001	4,058	59.41
Self mentions	1,084	17.53	<0.001	4,750	92.28	<0.001	8,320	121.81
Engagement markers	431	6.97	<0.001	1,089	21.16	<0.001	2,924	43.54
Total	10,891	176.16	<0.001	23,226	451.22	<0.001	37,268	545.61

CNEEC vs. CNEEC is a cross-linguistic comparison while CNEEC vs. ENEEC is a cross-cultural comparison under the current investigation.

Firstly, the frequency of transitions was consistent with the findings by Hyland (1998a) and Hyland and Tse (2004). As noted, the English-based sub-corpora, ENEEC and CNEEC, displayed an overall higher rate of transitions than the Chinese-based CNECC (268.44, 237.05 vs. 117.56 tokens per 10,000 words), and the cross-linguistic difference and the cross-cultural difference were both evidently significant at the 0.1% level. It is noteworthy that the Chi-square value between CNEEC and CNECC (2374.44) was far higher than that between ENEEC and CNEEC (116.41), suggesting that, for using transitions, Chinese ESL writers were more similar to their British-American counterparts than to Chinese L1 writers who were publishing in national journals.

Secondly, code glosses were significantly more frequently used in ENEEC and CNEEC than in CNECC (141.95, 136.28 vs. 63.63 tokens per 10,000 words). Both significant cross-cultural difference (ENEEC vs. CNEEC: *p* = 0.009) and cross-linguistic difference (CNEEC vs. CNECC: *p* < 0.001) were found. However, the Chi-square value in the latter (1548.95) was far higher than that in the former (6.86).

Then, frame markers were the least common interactive metadiscourse features in the three sub-corpora, whose frequencies were 32.43, 33.76 and 22.39 tokens per 10,000 words in ENEEC, CNEEC, and CNECC, respectively. Obviously, the cross-cultural influence of frame markers was not significant (*p* = 0.207). However, the gap was of statistical significance cross-linguistically (*p* < 0.001).

Next, endophoric markers were also more common in English-based sub-corpora (117.57, 116.82 and 58.79 tokens per 10,000 words in ENEEC, CNEEC and CNECC). The cross-cultural ENEEC-CNEEC difference was insignificant (*p* = 0.703), but the cross-linguistic CNEEC-CNECC difference was statistically significant (*p* < 0.001, Chi-square value = 1111.72).

Finally, there were also clear cross-cultural and cross-linguistic discrepancies between sub-corpora in the degree to which writers relied on prior literature as well as how writers presented these studies. Evidentials were 46.61, 64.93 and 31.70 tokens per 10,000 words in ENEEC, CNEEC, and CNECC, respectively. The cross-corpus comparisons were significantly different, i.e., *p* < 0.001 for ENEEC-CNEEC (Chi-square value = 181.65), and for CNEEC-CNECC (Chi-square value = 665.57). The data implied that scholars in the English-medium discourse community were more aware of drawing on supporting testimony and integrating new work into a discursive framework of accredited facts.

### Interactional metadiscourse

Generally, the vertical comparison showed that the distribution of interactional metadiscourse revealed similar tendency across three sub-corpora. Namely, in each sub-corpus, hedges occurred the most frequently, which were followed by boosters. Engagement markers contrastively occurred the least frequently ranging over the sub-corpora. Attitude markers overrode self-mentions in CNECC, but were overridden by self-mentions in CNEEC and ENEEC. On the other hand, the horizontal comparison verified that the frequency difference of interactional features between sub-corpora were cross-linguistically/cross-culturally significant (i.e., CNEEC vs. CNECC: *p* < 0.001, Chi-square value = 7275.75; and ENEEC vs. CNEEC: *p* < 0.001, Chi-square value = 545.36). British-American and Chinese scholars demonstrated markedly different persona in the academic texts when anticipating, acknowledging, challenging and suppressing potentially divergent positions. The findings were in consistency with the previous research (e.g., Hyland, 2004; Mu et al., 2015). Chinese ESL scholars demonstrated the same tendency of using interactional devices as the British-American counterparts although the former used significantly fewer interactional devices and expressed their cognitions and arguments in a more straightforward way.

Hedges constituted the largest main type of interactional metadiscourse in the three sub-corpora (198.18, 165.77 and 70.94 tokens per 10,000 words in ENEEC, CNEEC and CNECC respectively). The results were in line with previous studies where hedges held a dominant position, despite of genre, disciplines or languages involved (e.g., Moreno, 1997; Chen and Zhang, 2016). In fact, the frequency of hedges was only secondary to that of transitions in the current scheme, implying that expressing proper precision and new knowledge tentatively was of high necessity and importance in academic writing. The frequency Chi-square test run on hedges yielded significant main effects of both languages and cultures (*p* < 0.001). Still, the cross-linguistic effect (Chi-square value = 2240.60) far outperformed the cross-cultural effect (Chi-square value = 170.48).

The much higher frequency of hedges than boosters in QERAs (198.18 vs. 122.67 tokens in ENEEC, 165.77 vs. 107.70 tokens in CNEEC, and 70.94 vs. 46.46 tokens in CNECC per 10,000 words) indicated the emphasis on circumspection and discretion by academic discourse. Mitigation significantly exceeded accentuation in each sub-corpus (*p* < 0.001), which reflected both the significance of distinguishing facts from opinions in academic communities and the need to present claims provisionally other than assertively. Notably, the frequency Chi-square test run on boosters yielded significant main effects of both languages and cultures again (*p* < 0.001). Furthermore, the cross-linguistic effect (Chi-square value = 1429.14) far outperformed the cross-cultural effect (Chi-square value = 57.22). The significantly fewer use of boosters in CNEEC and CNECC indicated that Chinese scholars did not head off possible objections and take their own position, nor state their arguments as assertively and forcefully as their British-American counterparts. However, it was obvious that when Chinese ESL writers published internationally, their awareness of expressing certainty and constructing rapport rose significantly.

Then, the occurrence frequency of attitude markers was secondary to both hedges and booster in the current study (ENEEC: 59.41 tokens, CNEEC: 64.30 tokens and CNECC: 34.26 tokens per 10,000 words), which was in accordance with Ai’s (2012) and Thompson and Hunston’s (2020) arguments that epistemic assessment was more frequently utilized than attitudinal effect in academic texts. The cross-linguistic effect (Chi-square value = 531.78) was found to far override the cross-cultural effect (Chi-square value = 11.50). The significantly lower use of attitude markers in CNECC implied that Chinese scholars were more discreet when establishing their attitude towards data and inference in their native language and native culture. The overuse of these markers in CNEEC indicated that when they published in English, the confinement of expressing judgement explicitly melted with the euphemistic effect of a second language.

Contrastively, self-mentions were 121.81, 92.28 and 17.53 tokens per 10,000 words in ENEEC, CNEEC, and CNECC, respectively. The cross-corpus comparisons displayed significant differences (*p* < 0.001) for CNEEC vs. CNECC (Chi-square value = 3063.26), and for ENEEC vs. CNEEC (Chi-square value = 237.11). The giant gaps between sub-corpora reflected that scholars in the international academia were more aware of marking their roles in negotiating knowledge. The divergence was narrowed down between ENEEC and CNEEC, which implied that Chinese scholars were more conscious of accentuating their contribution in the international academia.

Lastly, there were significantly more engagement markers in English-medium sub-corpora (43.54, 21.16 and 6.97 tokens per 10,000 words in ENEEC, CNEEC and CNECC), and both the cross-linguistic and the cross-cultural divergence were significant (*p* < 0.001).

## Discussion

### Discoursal uniformities and context-situated strategies

Quantitative results in the previous section reveal that writers of QERAs use more interactive features than interactional features, and that metadiscourse strategies by English L1 scholars, Chinese ESL scholars and Chinese L1 scholars show distribution consistence to a large extent. In fact, the corpus under investigation demonstrate discoursal uniformities to a large extent.

As exemplified in (1) interactive metadiscourse signals the relationship between ideas and puts the materials in order so that readers could better understand and accept the arguments. It represents writers’ perception of readers and writers’ assumption of readers’ processing abilities and background knowledge. Explicitly, these devices lead readers through a discourse to each other and to related studies by ordering propositions. Among them, *transitions* link propositions by adding, comparing and explaining. As the most commonly used interactive metadiscourse features under discussion, they represent internal links in the discourse, and reflect the writer’s recognition of a need to help readers with an unambiguous recovery of an argument and logical reasoning (see (1a)). Then, *code glosses* are used to rephrase, explain, and illustrate what has been said (see (1b)). In RAs, statements are frequently supported by devices of propositional elaboration so that writers might promote understanding, guide readers to the preferred interpretation, and convey arguments in line with readers’ experience and knowledge (Hyland, 2007). Thereby, these features are primarily related to writers’ expectation and assessment of readers’ needs. Contrastively, *frame markers* provide framing information about text structure and frame elements of the discourse in a linear manner (1c). Text analysis presents that they are likely to occur in introductions where they help to specify the overall purposes and previous studies, and in discussions where they act to list reasons and explanations. Next, *endophoric markers* are reflexive items that refer to other parts of the discourse (see (1d)). They reflect authorial assessment of the discourse and readers, and connect propositions by navigating the to-and-fro between visual and verbal elements. Finally, *evidentials* present the source of information and community-based literature, and provide justification for propositions by citing or attributing (see (1e)). They contribute to not only providing justification for arguments and establishing writers’ credentials and novelty, but demonstrating an allegiance to the research conventions and the academic community. Finally,

(1a)**However**, some sellers may explicitly prefer that…(ENEEC-35)
(1b)**For example**, scholars have examined…(CNEEC-22)
(1c)To fill this knowledge gap, **the primary objective of this paper is to** explore…(CNEEC-18)
(1d)First, **as mentioned earlier**, Shanghai Stock Exchange (SHSE) and Shenzhen Stock Exchange (SZSE)…(CNEEC-26).
(1e)**Ang et al. (2015)** proposed a multi-regional (M-R) spatial decomposition model…(CNEEC-19)
(2a)**On average**, the growth rate is faster…(CNEEC-4)
(2b)China has **always** been promoting the implementation of…(CNEEC-19)
(2c)Rather, it is more **important** and **interesting** to understand…(CNEEC-21)
(2d)**My** approach in this article builds on the premise that…(ENEEC-46)
(2e)**Note that** a depreciation of the yen, euro, etc. simultaneously leads to…(ENEEC-36)

As illustrated in (2), interactional type is particularly critical in manifesting how writers intrude into the text so as to imply their interpretations of both the message and the readers. Among them, *hedges* are prevalent and essential in academic writing in view of their functions in presenting tentative propositions and proposing possibilities with “[caution] and precision” (Hyland, 1996, p. 433). Usually, hedges are used to reduce the force of statements and express uncertainty, skepticism, humility and deference involved in RAs (see (2a)). But scholars are not always so accommodating and conciliatory, and they would not be so convincing if they were. Instead, they use *boosters* to underline the conviction, to mark solidarity, and to direct engagement with readers in text construction (see (2b)). Then, *attitude markers* imply writers’ affective or evaluative attitude to propositions (Hyland, 2005). Conveying surprise, (dis)agreement, significance, obligation or frustration, they play an important role in enhancing the persuasiveness of arguments with shared value system and attitude. What’s more, with attitude markers, writers might further claim solidarity with readers and peers since these labels are reflections of social values and norms of a particular discourse community (see (2c)). In contrast with the implicitness of attitude markers, *self-mentions* are mostly explicit features of authorial identity in the text. In other words, they are writers’ self-references and self-citations which allow their direct projection into the discourse to illustrate their identities explicitly. As argued by Hyland (2001b), self-mentions are multifunctional in RAs. Not only do they organize the discourse, but the present or absent reference of authorship is the writer’s conscious choice to indicate his/her stance as well as his/her linguistically-situated and culturally-specific identity (see (2d)). As a result, self-mentions fulfil an efficient rhetorical function to highlight writers’ contribution and mark their role in negotiating propositions and claims. While other types of interactional metadiscourse are writer-oriented and indicative of ways writers present themselves and commit themselves to claims, *engagement markers* are reader-oriented and concerned about ways writers recognize the presence of readers and include them as intelligent participants (Hyland, 2001a; see (2e)). They are devices that “[explicitly] address readers, either to focus their attention or to include them as discourse participants, and therefore accentuate persuasiveness in knowledge construction” (Hyland, 2005, p. 53).

At the same time, however, sinigicant frequency discrepancies are found both cross-linguistically and cross-culturally. Results Section shows that metadiscourse features are significantly more present in the QERAs written in English than in Chinese, and markedly more present in those by scholars based at British-American institutions than in those by scholars based at Chinese institutions. The varied metadiscourse strategies are reflections of distinctively-preferred linguistic choices and textual developments by different linguistic/cultural communities. These differences prove that writers speaking different languages or from cultural communities are aware, to different degrees, of readers and their need for elaboration, clarification, guidance and interaction.

Despite of the presence of both the cross-linguistic and the cross-cultural gaps, Chinese ESL scholars, in most cases, are found to be more similar to their British-American counterparts with regards to metadiscourse strategies. Thus conclusions could be drawn that Chinese ESL writers have basically developed academic awareness to use more metadiscourse devices in QERAs though their native language and native culture have been influencing their ESL language choice, text-organization, communication, and to be more specific, their perception of readers. Rhetorical choices and metadiscourse strategies available to them are shaped by values and conventions prevailing in the linguistic/cultural community where the text is written/published. In short, language-specific and culture-specific patterns of writing actually cause interference at the discursive level in their ESL academic writing.

### Correlated metadiscourse and metacognition

Language is an integral part of human cognition (Langacker, 1987), and arguably, metalanguage and metadiscourse are organic parts of metacognition (Tang, 2021). While metalanguage concerns our knowledge about language, metadiscourse embraces discourse-monitoring and interpersonal functions (Hyland, 2017). Contrastively, metacognition is the advanced monitoring system in the cognitive processing, and it plays its role in every specific cognitive domain (Li, 2003). Metadiscourse and metacognition interact with one another in the verbal communication in which linguistic information is encoded and decoded, and meanings are constructed and reconstructed. This process is unavoidably influenced by metacognition which is responsible for establishing communicative goals, monitoring understandings, mediating the appropriateness of discourse, guaranteeing the fluency of communication and so on.

(3) …are required to match **at least** 20% of…**and must** meet…is **generally** limited to…, **though**…**may** be eligible **if**…, **such as** participation in the Temporary Aid for Needy Families (TANF) program.

As illustrated in (3), the writer consciously deploys transition markers *and, though* and *if*. Without them, readers might finally figure out the causal-effect/concession/condition-result relationship despite paying more cognitive efforts. With them, however, readers might immediately understand the logical relationship with the least degree of cognitive efforts, and that’s why the writer applies these markers initiatively. In a similar fashion, writers should calculate the weight to be given to his/her propositions, anticipate the degree of propositional precision or reliability, and hence indicate readers the epistemic status of propositions either as accredited facts or authorial interpretation. So, when using hedges *at least, generally* and *may*, the writer presents information as an opinion rather than a fact, and s/he accordingly suggests that claims in the stretch of the discourse are grounded in his/her credible reasoning instead of specific knowledge. Then, the writer uses *must* to imply his/her affective attitude and make sure readers would understand the obligation of this situation. Besides, the writer resorts to the code gloss *such as* to ensure readers are able to recover his/her intended meanings, and the parentheses to explain a previous discourse unit. Both of them are suggestive of the writer’s prediction about readers’ knowledge background. Therefore, these metadiscourse strategies are reflections and labels of writers’ cognitive and metacognitive activities by which writers could, consciously and unconsciously, organize the discourse and interact with readers. The previous section points out distribution consistence of metadiscourse in the sub-corpora, which can be explicated in terms of disciplinary norms and generic conventions that curb writers’ metacognition regarding metadiscourse strategies. On the other hand, academic texts in English show greater tendency for explicit structure and purposes, less tolerance of digressions, more caution in making claims, and higher frequency of using sentence connectors. The cross-linguistic and cross-cultural variances in using metadiscourse can be attributed to both cultural and cognitive explanations as follows.

Some aspects of metadiscursive discrepancies might be explicated in social-cognitive terms since divergent discourse paradigms are valued in various rhetorical traditions and across languages/cultures. Take self-mentions for example. The variances between British-American scholars and Chinese scholars in using *I*, *we*, *my*, and *our* largely reflect value differences—collectivism and individualism (Hofstede et al., 2010). British-American scholars apply more singular self-mentions such as *I* to actively present themselves and engage in claims or propositions. Their frequently explicit appearance in a text contributes to creating a plausible academic identity and constructing their persona with which they are to present an argument. In this case, British-American scholars cherish their culture-specific individualism which advocates independent self-construal (Markus and Kitayama, 1991), underlines active self-realization, and stresses direct presentation of attitude, so they could draw themselves closer to readers and make themselves sound more reliable. Contrastively, Chinese writers make use of much fewer *I* in argumentation, and appear to misrepresent their own ideas or claims as general agreement or truth. At the same time, by the frequent use of *we* instead of *I*, Chinese writers succeed in assuming shared understanding, mixing themselves with others, mitigating their own individual identity, and finally creating a collectivized voice. Admittedly, this strategy suppresses their voice, de-recognizes their identity, and stops the due promotion of their professional persona (Abbas and Shehzad, 2018). However, as Chen (2020) proposes, the motivation for Chinese writers to avoid the first person singular pronoun for single-author self-reference is modesty, i.e., a need to mitigate authority inherent in academic authorship. That is, Chinese scholars tend to construct their identities as collective, and prefer distancing themselves from being authoritative with third person NPs and inanimate devices. In their academic culture, authorship entails authority, and being authoritative runs against the culturally-rooted notion of modesty.

On the other hand, the great metadiscursive divergence cross the sub-corpora can be explored in cognitive terms. As noted previously, Chinese scholars use significantly fewer transitions, code glosses, frame markers, and endophoric markers in their L1 discourse organization. In effect, they are more dependent on topic chains that operate between the run-on sentences to connect the discourse and organize the context. Seemingly, Chinese run-on sentences and English complex sentences are formally and functionally alike. But as Zhao and Wang (2020) claim, they are fundamentally different from each other as for their subject-predicate structure, attribute and relationship of sentential components, use of cohesive devices, boundary of sentence and even the punctuation. While the former represents the strong spatiality of Chinese syntactical structure, the latter reveals the pro-temporality of English discourse. With the mutual influence of languages and thoughts, Chinese L1 writers extend the texts and construct the topic chains based on their unique cognitive structures. Once the topics are fixed, they need not care much about the syntactic structures between adjacent clauses. Instead, they are more dependent on topic chains to connect the run-on sentences or clauses by the means of zero anaphora, zero cataphora and other cohesion patterns, and to extend discreteness and parataxis of the discourse. Therefore, their L1 discourse largely results in being topic-prominent, and their uses of interactive metadiscourse are far less frequent. Furthermore, their spatiality preference of discourse conceptualization in this sense extends its influence, and reveals L1-based transfer in their ESL writing, which can mostly explicate the significant gaps between ENEEC and CNEEC.

Tang (2021) points out close connection between metadiscourse and metacognition which frequently overlap and manifest concurrently. As contended by Guillem (2009), arguing is not a purely internal process occurring within speakers’/writers’ minds and hence unable to be observed. Rather, a great many phenomena conventionally categorized as mental processes are essentially formed within the discourse (Billig, 2003). In fact, when deciding on a particular metadiscourse device, writers strategically pick up an appropriate form for communication at the moment. For example, when expressing affective and evaluative attitude to propositions, writers may resort to *interesting, surprising* and *important*; when emphasizing certainty and marking involvement with the claims, writers may use *believe, in fact* and *definite*; and when directing readers to act or to see particular items, writers may use *see, refer to* and *take … for example*. Therefore, it is worthwhile to examine argumentation in RAs as a process in which writers make use of their knowledge both in the form of their personal beliefs and the shared attitudes. As Shi (2014) notes, the interpersonal capability of metacognition is in effect metadiscourse competence, and the interaction of metacognition between speakers/writers and listeners/readers is the cognitive mechanism of metadiscourse. On account of the interpersonal communication of metacognition between writers and readers in QERAs, this paper preliminarily concludes that the metadiscourse strategy is a linguistic reflection of metacognition competence, and that metacognition controls and monitors the process of communication partly by the means of metadiscourse devices. In view of the proposal that Chinese ESL writers demonstrate discursive transfer of metadiscourse strategies, this study further argues that such transfer is conceptualized transfer in that it happens within the domain of metacognition and cognition.

### A model of correlated metadiscourse and metacognition

**Figure 2:**
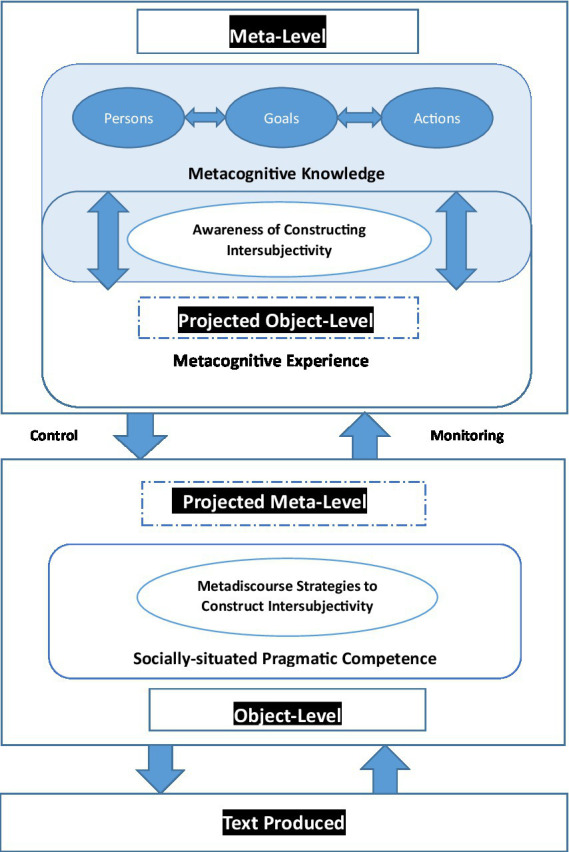
A model of correlated metadiscourse and metacognition in writing RAs.

Owing to Flavell’s (1979) Model of Cognitive Monitoring and Hacker’s et al. (2009) Metacognitive Model of Writing, this study suggests a Model of Correlated Metadiscourse and Metacognition in Writing RAs (see Figure 2). It proposes that at meta-level, British-American scholars and Chinese scholars apply metacognitive knowledge, undergo metacognitive experiences, accomplish goals and implement strategies in culturally/linguistically different ways though they exhibit shared disciplinary/generic awareness to a certain degree. In this Model, cognitions at meta-level and object-level occur concurrently—scholars write a text and construct its content (i.e., an object-level process) when thinking about whether his/her propositions or interpretations are accurate or reasonable (i.e., a meta-level process). At the meta-level, metacognitive knowledge and metacognitive experience which are involved in writers’ awareness to construct intersubjectivity play different roles but overlap with one another in writers’ metacognitive activities. At the object-level, writers compose the texts and construct intersubjectivity with metadiscourse strategies. The writing process simply involves more strategies than the metadiscursive ones; but the current Model is focused on metadiscourse strategies, and it suggests that metadiscourse strategies co-occur with other strategies in the writing process where strategies concerned are interwoven with each other both horizontally and vertically, and both hierarchically and parallelly.

#### At the meta-level

This Model keeps Flavell’s (1979) differentiation between metacognitive knowledge and metacognitive experience in addition to major categories of these variables—person, task, and strategy. Firstly, the *person* category involves everything that writers could come to believe about the nature of themselves and readers as cognitive processors. It can be further categorized into beliefs about intra-individual differences, inter-individual differences, and universals of cognition. An example of intra-individual differences is that some readers of RAs could understand information better with the aid of tables and graphs than with non-visual information such as those in paragraphs. An example of inter-individual differences is that some readers could better understand than others the logical relationships between propositions with or without explicit metadiscursive features such as transitions, frame markers or endophoric markers. An example to illustrate universal properties of cognitions is that writers are aware of potentially various degrees and kinds of understanding by readers, or is that writers and readers are basically dependent more on transitions in organizing/understanding the discourse other than code glosses, frame markers, endophoric markers or evidentials.

Secondly, the *goal* (or *task*) category concerns the information available during a cognitive enterprise or the information regarding task goals/demands. The metacognitive knowledge in the former case might be both the understanding of various kinds of information and the awareness of how the cognitive enterprise (i.e., to complete the writing process and the presentation of information and opinions) should best be managed in order to achieve the goals. In contrast, the metacognitive knowledge in the latter case could be task goals or demands on account of which some cognitive enterprises are found much more difficult or easier than others. In the case of the current research, writers’ metacognitive knowledge is interwoven with their expectation of readers’ metacognitive knowledge. Thus, writers have the realization, albeit to different degrees, that information provided in RAs might be understood as familiar or unfamiliar, abundant or insufficient, demanding or easy, trustworthy or untrustworthy and so on.

Thirdly, the *action* (or strategy) category is related to what strategies tend to be effective in fulfilling tasks or goals of cognitive enterprises among the huge repertoire of acquired knowledge. Such metacognitive strategies are typically reflected in the employment of particular metadiscourse strategies under investigation. That is, writers of QERAs, based on their judgement of readers’ metacognitive knowledge, cognitive knowledge and possible doubts or questions, should resort to, for example, code glosses to clarify their intentions or to illustrate their propositions. Obviously, their judgment is language-specific and culture-specific to a significant degree.

Most metacognitive knowledge essentially entails interactions of these three variables in writers’ construction of intersubjectivity: *persons* concern both writers and writers’ judgement about readers; *goals* mean the task to clarify intentions or to illustrate propositions; and *actions* are writers’ decision to employ metadiscourse features such as code glosses. The interrelatedness of these variables are indicated by the bilateral arrows between them. In this case, writers’ metacognitive knowledge and awareness to construct intersubjectivity are activated deliberately by a search for effective metadiscourse strategies, or otherwise are activated unintentionally by retrieval cues in the goals-context. Their metacognitive knowledge, presented in the form of declarative, procedural and conditional knowledge (Schraw, 1998), thus gives rise to a variety of metacognitive experiences, and then helps them to understand the implication of these metacognitive experiences.

By contrast, metacognitive experiences, which are “[experiences] concerning self, tasks, goals, and strategies” as well as the results of activated metacognitive knowledge (Flavell, 1979, p. 908), can occur at any time before, after, or during the cognitive enterprise of writing RAs. For example, writers may feel that they are not adequately communicating to readers what they believe, agree with or doubt about. Or at other times, they may feel suddenly stuck in an attempt to present arguments that they are addressing. Flavell predicts that metacognitive experiences are more likely to occur when situations stimulate careful, highly-conscious thinking. Specifically, some metacognitive experiences are simultaneously items of metacognitive knowledge which have entered authorial consciousness while others are not. It follows that metacognitive knowledge and metacognitive experiences partially coincide, which in turn may or may not have entered authorial consciousness to construct intersubjectivity. As stated, metacognitive experiences may have critical impact on metacognitive knowledge by revising goals or by activating strategies. In fact, the bilateral arrow in the overlapping area of the Model indicates the interaction between metacognitive knowledge and metacognitive experience.

On one hand, writers of QERAs may give up old goals and come up with new ones due to the possible setbacks in the process of researching and writing. On the other hand, their metacognitive experiences can activate strategies to deal with cognitive and metacognitive goals. Chien (2006) argues that cognitive strategies are strategies designed to solve problems while metacognitive strategies are utilized to monitor, evaluate, control and understand these cognitive strategies. Take the writing process of RAs for example again. Writers may feel (i.e., metacognitive experience) that they do not yet know well enough a certain theory cited in their articles to support their claims and convince their readers, so they read the theory again and refer to more relevant literature (i.e., cognitive strategy, aimed at the cognitive goal of improving their knowledge). Contrastively, writers may wonder (i.e., metacognitive experience) whether they know well enough a certain theory cited in their articles to support their claims, so they ask themselves questions about it, presuppose potential doubts from the perspectives of readers, and then specify how well they can answer them (i.e., metacognitive strategy, aimed at the metacognitive goal of assessing their knowledge). Namely, metacognitive knowledge is inclined to contain knowledge of both metacognitive strategies and cognitive ones; alternatively, the same strategy may be invoked for both metacognitive and cognitive goals.

The monitoring of cognitive enterprises performs through the actions of as well as proceeds with the interactions among metacognitive knowledge, metacognitive experiences, goals (or tasks), and actions (or strategies). This dynamic interplay system could be summarized with another hypothetical example. Once goals or tasks are established, writers’ existing metacognitive knowledge concerning these goals gives rise to conscious metacognitive experiences—these goals might be a big challenge for them. These metacognitive experiences, together with additional metacognitive knowledge, propel writers to apply the cognitive strategy of resorting to previous literature or established theories. The outcome of the resorting triggers supplementary metacognitive experiences about how the efforts might proceed. The metacognitive experiences that are guided by appropriate metacognitive knowledge would then activate the metacognitive strategies to reflect on what writers have learned. Writers can see whether new arguments or presumptions are consistent with authorial prior knowledge and expectations, whether they are comprehensible to readers, and whether they provide an avenue to goals. The interplay of metacognitive knowledge and experiences of cognitive and metacognitive strategies keeps going on until it brings the metacognitive enterprise to a close.

#### At the object-level

Yet the metacognitive enterprise of writing related with metadiscourse is not completed at the meta-level. The object-level including cognitions about external stimuli or internal stimuli is connected with the meta-level through control and monitoring. The current Model is in line with Hacker et al. (2009) in that the meta-level controls the object-level in order to produce thoughts whereas the object-level monitors the meta-level so that the production of thoughts can be observed.

As argued, metadiscourse strategies are formed at the object-level as the intersubjectivity-constructing resources. In this case, metacognitive monitoring takes place at the object-level by a flow of information from the object-level to the meta-level, and then the meta-level is informed about metadiscourse cognitions (i.e., the need and the rationality to organize the discourse and interact with readers in specific community). The metadiscourse cognition gives rise to the construction of a dynamic model of the object-level at the meta-level (Conant and Ashby, 1970; Hacker, 2018). This paper contends that by the means of monitoring, the projected object-level in the meta-level activates writers’ awareness of constructing intersubjectivity which occurs among the interplay system between metacognitive knowledge and metacognitive experience. The meta-level now contains mental representations of *persons* involved (e.g., writers and readers), *goals* to be accomplished (e.g., to explicitly point out the logical relationship) and *strategies* that meta-level can use the object-level to accomplish them (e.g., the use of transitions, boosting and hedging).

On the other side of this Model, metacognitive control occurs “[through] a flow of information from the meta-level to the object-level that is intended to guide and direct object-level cognitions” (Hacker, 2018, p. 2). The modification of the metadiscourse metacognition results in the projected dynamic meta-level at the object-level. This study further predicts that by the means of information-control, the projected meta-level at the object-level initiates, modifies or cancels particular patterns of writer-reader-communication, goals to be fulfilled and metadiscourse strategies to be applied. The interplay system of three variables (i.e., *persons*, *goals* and *actions*), in addition to the interplay system of metacognitive knowledge and metacognitive experiences, interacts with the object-level continuously before, during and after the writing process. The modified thoughts are supposed to be recycled through additional cognitive monitoring which, in turn, would update the projected object-level at the meta-level. On one hand, information-control informs writers of whether interactive or interactional metadiscourse strategies are well used or poorly employed, for example, to clarify writers’ ideas or to establish writers’ explicit/implicit identities. On the other hand, it informs writers of whether further metacognitive control needs to be used to make additional changes to the metadiscourse strategies. Therefore, the simultaneous flow of metadiscourse information between meta-level cognition/metacognition and object-level cognition allows writers of RAs to initiate and modify their thoughts on metadiscourse as well as to observe those metadiscourse strategies as intersubjectivity-constructing resources. To be more specific, knowing what, how, when and why to use different metadiscourse devices towards achieving particular goals contributes to the cognitive development of metadiscourse strategies. Again, this process involves linguistic/cultural specificity as well as disciplinary/generic uniformity.

No matter what metadiscourse strategies writers bring to their minds at the object-level, the thoughts must be reflected at the meta-level through monitoring and control. Take transitions for example. Writers can control explicitly additional, contrastive or consequential relationships at the object-level only if they first have knowledge of syntax/discourse and meta-level knowledge/experience of the way syntax/discourse can be controlled. This control process can be further developed if writers have a meta-meta-level knowledge/experience of syntax/discourse. In other words, writers possess metacognitive knowledge/experience concerning the way syntax/discourse can be controlled, and they have metacognitive knowledge/experience of that knowledge/experience. Take engagement markers for another example. They can be controlled at the object-level, and they observe the production of writers’ thoughts at the meta-level. The production of writers’ thoughts at the meta-level should involve writers’ metacognitive and meta-metacognitive knowledge/experience regarding writer-reader communication (e.g., the significance of involving readers and the way to include readers’ participation). Only in a cycling way can the controlled and monitored processes keep going on and being enhanced, and finally contribute to the text produced. To sum up, this study proposes that if the meta-level knowledge/experience concerning metadiscourse strategies undergo a more complete and elaborate process, writers could have greater potential to control the extent and quality of metadiscourse devices which finally contribute to discourse organization and writer-reader interaction in the texts.

Then, how do the socially-situated metadiscourse strategies construct intersubjectivity as a part of socially-situated pragmatic competence at the object-level? Hacker (2018) claims that when phonology, syntax, semantics and pragmatics become objects of investigation, they can be represented at higher levels of representation as metaphonology, metasyntactics, metasemantics and metapragmatics. In view of their pragmatic attributes, metadiscourse strategies can find their position in the meta-level as one part of metacognitive knowledge/experience. Namely, metacognitive knowledge/experience entails metapragmatic/metadiscourse knowledge/experience. Metapragmatic knowledge is different from other constituents of a language because it is a clear manifestation of one’s metalinguistic experiences as a whole including other important elements of language (van Kleeck, 1984). While metapragmatic knowledge refers to knowledge of the rules for using language, metapragmatic experience includes all of one’s metalinguistic experiences as well as all of one’s knowledge of language and its uses (Gombert, 1993). Gombert’s differentiation endorses the following claims: (1) metadiscourse knowledge, as a part of metapragmatic knowledge, includes knowledge of *persons* (e.g., readers and writers as participants), knowledge of *tasks* to be accomplished by metadiscourse (e.g., to cite classic research to establish more credibility) and knowledge of *strategies* to be used to achieve a particular goal (e.g., to explicitly/implicitly mention writers themselves to show their intended identities); and (2) metadiscourse experience, as a part of metapragmatic experience, includes all of writers’ metadiscourse knowledge and its uses. So far, writers’ awareness of constructing intersubjectivity at the meta-level has been formally realized at the object-level as metadiscourse strategies. The previously-mentioned metadiscourse variations reflect that authorial awareness of intersubjectivity-construction varies to a large extent due to linguistically-situated and culturally-specific effects.

As expected, the metapragmatic awareness is located at the meta-level correlation between metadiscourse and metacognition in writing RAs. It is self-monitoring and is of reflectivity. The presence/absence of metadiscourse is one of the most obvious and convenient ways to demonstrate the reflectivity. The reflexivity of writers, or the metapragmatic awareness of writers, is manifested at two levels in this case. The first is the writer-text level which is realized by interactive metadiscourse devices; it is here that writers construct and constrain the discourse in accordance with anticipated needs of specific readers. The second is the writer-reader level that is realized by interactional metadiscourse devices; it is here that writers evaluate the statements and sing their voices in line with cultural conventions and norms. Both of the reflexivity levels tell that metadiscourse strategies are results of on-going adaption and linguistic choices in diverse contexts by interlocutors whose metadiscourse awareness may dynamically vary to different degrees of salience. That’s why Ran (2005) argues that metadiscourse strategies are reflective of one’s metapragmatic awareness and metapragmatic competence. The two concepts are similar to each other to a certain degree in that both of them are contextually-situated, generically-specific and culturally-determined, and in that both of them encompass writers’ appropriate use of linguistic devices in order to construct their identities and align themselves with readers in their RAs. The interwoven relationship between metadiscourse and metapragmatics/metapragmatic awareness is typically suggestive of the pragmatic nature of metadiscourse. So far, it could be concluded that metacognition and pragmatic competence are embodied in metadiscourse strategies, and accordingly, metadiscourse strategies are included as a part of socially-situated pragmatic competence at the object-level in the current Model.

To summarize, academic writing entails writers’ attempt to balance their own goals with readers’ expectations through a process of negotiation. Writers select their language to engage readers and to convey their messages which make most sense to readers. Besides demonstrating authorial construal of the world and presenting transmission of knowledge, writing is an interactive and dynamic process of recognizing, constructing and negotiating between writers and readers in culturally-situated and disciplinarily-specific context. Taking into consideration readers’ orientation, interest, knowledge, need and capability of understanding and analyzing, reader-oriented writers organize the discourse and interact with readers as effectively as possible with linguistically-related and culturally-related metadiscourse strategies. Cross-linguistic and cross-cultural variations that are previously-presented manifest, to a diverse extent, writers’ anticipation of readers’ responses to relevant assumptions as well as their attempts to build up authorial identities as insiders. Both British-American writers and Chinese writers display their familiarity with the practices of their current discourse communities. They encode information, implement warrant and structure arguments in the way their readers may find most convincing in their respective native culture. With previous experience in addressing academic discourse, they predict how readers would respond to their arguments, what readers may find convincing or doubt about, and where readers are likely to need elaboration. As a result, the process of reader evaluation helps writers to construct their reasoning—reasoning in analyzing the context, reasoning in presenting information, reasoning in communicating with readers, and reasoning in using appropriate language.

RAs are produced within the disciplinary community. Whether writers’ arguments could be accepted or not is largely dependent on the deliberate and effective manipulation of writers upon the interaction between writers and readers (Hyland, 2005). In order to construct the discourse of interaction, writers not only make a balance between expressing their own beliefs and addressing readers’ need, but also abide by disciplinary conventions and social values of a specific disciplinary community. On the other side, readers expect that writers would organize the discourse, express arguments, and show their persona by reasonably using interactive and interactional metadiscourse devices in the written interaction. Thereby, writers might not convince readers nor construct knowledge until they have made valid prediction about readers and about readers’ possible responses. In a word, the whole process of knowledge construction thrives on writers’ awareness of intersubjectivity in the context and their realization of using metadiscourse to accomplish intersubjectification in the context.

## Conclusion

This paper compares metadiscourse strategies by English L1 scholars, Chinese ESL scholars and Chinese L1 scholars. It finds that British-American scholars use more metadiscourse devices than Chinese scholars, and that Chinese ESL scholars use more metadiscourse devices than Chinese L1 scholars. Chinese ESL writers demonstrate influences of their native language and native culture at the discursive level, and reveal L1-based discursive transfer in employing these devices. In addition to cultural motivations, the cognitive implications of culture-specific and language-specific metadiscourse variations are discussed in terms of the correlation between metacognition and metadiscourse. And the paper further proposes that metadiscourse is the linguistic reflection of metacognition, and that metacognition exerts mediation and monitoring over cognitive objects partly by the means of metadiscourse.

The corpus under investigation is restricted to a small number of RAs in Economics in 2010s, but metadiscourse strategies might vary across disciplines and develop across time. Thus, the results cannot be extended to the whole academic culture and should be taken with caution. Future research, however, can comment on whether rhetorical similarities and differences revealed in this study are relevant to other disciplinary communities. Note that metadiscourse in any coding scheme can hardly achieve a comprehensive description. Future studies should go beyond texts and corpora, and draw on more sources of evidence such as interviews with the insiders, expert self-reports and so on. Such a triangulation method would help us to better understand how insiders write and respond to metadiscourse features.

ESL scholars have been showing an increasingly stronger tendency to participate in the relevant disciplinary communities largely through writing RAs (Abdi et al., 2010), but a large number of rejections of their articles by international scholarly journals are said to be due to linguistic and non-linguistic unsophistication. Along these lines, ESL scholars should be more aware that effective argument and linguistic appropriateness are indeed contextually-situated and community-oriented. They need to understand the distinctive way their discipline operates in addressing peer experts and presenting arguments. Therefore, studies like the current one are expected to promote writers’ awareness of possibly varying values and contrastive conventions in domestic and international discourse communities. However, it is critical to point out that a particularly preferred way of connecting ideas or engaging readers in a specific setting is simply not more sophisticated or advanced than others in the cognitive sense.

## Data availability statement

The original contributions presented in the study are included in the article/supplementary material, and further inquiries can be directed to the corresponding author.

## Author contributions

All authors listed have made a substantial, direct, and intellectual contribution to the work and approved it for publication.

## Funding

This research was supported by the National Education Sciences Planning Projects in China (DIA190396).

## Conflict of interest

The authors declare that the research was conducted in the absence of any commercial or financial relationships that could be construed as a potential conflict of interest.

## Publisher’s note

All claims expressed in this article are solely those of the authors and do not necessarily represent those of their affiliated organizations, or those of the publisher, the editors and the reviewers. Any product that may be evaluated in this article, or claim that may be made by its manufacturer, is not guaranteed or endorsed by the publisher.
